# Open Science Practices in Gambling Research Publications (2016–2019): A Scoping Review

**DOI:** 10.1007/s10899-022-10120-y

**Published:** 2022-06-09

**Authors:** Eric R. Louderback, Sally M. Gainsbury, Robert M. Heirene, Karen Amichia, Alessandra Grossman, Bo J. Bernhard, Debi A. LaPlante

**Affiliations:** 1 Division on Addiction, Cambridge Health Alliance, a Harvard Medical School Teaching Hospital, Malden, MA USA; 2 Harvard Medical School, Boston, MA USA; 3 University of Sydney, Sydney, Australia; 4 International Gaming Institute, University of Nevada, Las Vegas, NV USA; 5 University of Nevada, Reno, NV USA

**Keywords:** Gambling, Open science practices, Pre-registration, Gambling disorder, Problem gambling, Scoping review

## Abstract

The replication crisis has stimulated researchers around the world to adopt open science research practices intended to reduce publication bias and improve research quality. Open science practices include study pre-registration, open data, open access, and avoiding methods that can lead to publication bias and low replication rates. Although gambling studies uses similar research methods as behavioral research fields that have struggled with replication, we know little about the uptake of open science research practices in gambling-focused research. We conducted a scoping review of 500 recent (1/1/2016–12/1/2019) studies focused on gambling and problem gambling to examine the use of open science and transparent research practices. Our results showed that a small percentage of studies used most practices: whereas 54.6% (95% CI: [50.2, 58.9]) of studies used at least one of nine open science practices, each practice’s prevalence was: 1.6% for pre-registration (95% CI: [0.8, 3.1]), 3.2% for open data (95% CI: [2.0, 5.1]), 0% for open notebook, 35.2% for open access (95% CI: [31.1, 39.5]), 7.8% for open materials (95% CI: [5.8, 10.5]), 1.4% for open code (95% CI: [0.7, 2.9]), and 15.0% for preprint posting (95% CI: [12.1, 18.4]). In all, 6.4% (95% CI: [4.6, 8.9]) of the studies included a power analysis and 2.4% (95% CI: [1.4, 4.2]) were replication studies. Exploratory analyses showed that studies that used any open science practice, and open access in particular, had higher citation counts. We suggest several practical ways to enhance the uptake of open science principles and practices both within gambling studies and in science more generally.

**Supplementary Information:**

The online version contains supplementary material available at 10.1007/s10899-022-10120-y.

## Introduction

Many behavioral science research fields have surprisingly low likelihoods of replicating key findings (Camerer et al., 2016, 2018; Nosek & Errington, 2020; Nosek & Lakens, 2014; Open Science Collaboration, 2012, 2015) and struggle with publication bias (i.e., a preference for publishing statistically significant results; Anderson et al., 2017; Ferguson & Heene, 2012). Research stakeholders have proposed that using open science principles and practices, along with study replication, is one way of combating these threats to research quality and accuracy (e.g., Blaszczynski & Gainsbury, 2019; LaPlante, 2019; LaPlante, Louderback, & Abarbanel, 2021; Louderback, Wohl, & LaPlante, 2021; Munafò, 2016). Yet, recent scoping reviews of behavioral research generally and the substance-related disorders literature in particular have indicated that the use of practices such as pre-registration, open materials, open data availability, and more, has been limited (Adewumi, Vo, Tritz, Beaman, & Vassar, 2021; Hardwicke et al., 2021). Likewise, direct replications of published research are rare. For example, a substance-related disorders (e.g., opioid use disorder) scoping review study (i.e., Adewumi et al., 2021) observed only one replication study in a sample of 300 studies published during the 2014–2018 period.

Although gambling studies^1^ shares methods, study instruments, and theoretical perspectives with other types of behavioral research, including research on substance use disorders, information about the use of contemporary open science research practices in gambling studies is very limited (see LaPlante et al., 2021). Accordingly, in this article, we report the outcomes from a scoping review of a random sample of 500 studies about gambling-focused topics and systematically map the existing gambling research and identify gaps in open science research practices. In completing this review, we have provided new information about the nature of gambling-related research practices and potential practice deficits that risk a low likelihood of replication and a high likelihood of publication bias. This information is central to estimating the quality and methodological rigor of the published literature related to gambling and for providing clear recommendations for developing a high-quality, replicable research literature that can effectively inform evidence-based policy.

## Background

### Open Science Principles and Practices

Open science practices have grown in popularity in scientific research during the past decade (Banks et al., 2019; Nosek & Lindsay, 2018). This rise in interest and use of open science practices came in response to two major events in science: (1) the identification of a “replication crisis” in multiple disciplines, most notably in psychology, characterized by low success rates when replicating previously significant results on different samples (Klein et al., 2018; Lindsay, 2015; Loken & Gelman, 2017; Maxwell et al., 2015; Schooler, 2014), and (2) widely-publicized examples of fabrication and falsification of data in high-profile published scientific research (Callaway, 2011; Eggertson, 2010; Godlee, Smith, & Marcovitch, 2011; Schulz et al., 2016; Wicherts, 2011). Following these two events, scholars took steps to enhance the transparency, rigor and validity of scientific research by developing and promoting open science practices, including research pre-registration and Registered Reports, separation of confirmatory and exploratory analyses, open data, open materials and open access (e.g., Nosek & Lakens, 2014; Nosek et al., 2018; West, 2020). Each of these practices is encompassed under the larger umbrella of “open science” practices.

First, research pre-registration refers to a detailed public and time-stamped document containing research questions, hypotheses, study methods, and plans for analysis that is written before any data collection or analysis takes place. These documents are typically registered online through an independent organization, such as the Open Science Framework or a clinical trials registry (e.g., clinicaltrials.gov). Pre-registrations are usually made public, yet can be embargoed for a period of time (e.g., 6 months) to prevent the possibility that another researcher might “scoop” an idea before the original team is able to publish the results or to ensure that journal requirements for blinded submission are met.

Second, open science explicitly differentiates between confirmatory and exploratory analyses. Confirmatory analyses are pre-registered, based on a pre-planned design for analysis created before the data was collected with the aim of testing specific hypotheses about the sample(s). When developing confirmatory hypotheses, best practices for research suggest that an *a priori* power analysis should be carried out to determine the necessary sample size to detect statistically significant results (see O’Keefe, 2007). Exploratory analyses should be sufficiently powered; however, they are not pre-registered and consist of testing relationships among variables in the data that were not pre-planned. Exploratory analysis can provide the basis for future, pre-registered research that tests confirmatory hypotheses.

Third, open data pertains to making data available to the public in a freely accessible location with limited steps to access it. Providing data to the public increases transparency within research, allows independent observers to engage in reproducibility checks of published findings, and might encourage researchers to conduct novel independent or collaborative secondary analyses. In addition, open data can stimulate meta-scientific research that analyzes multiple datasets in systematic reviews and meta-analyses (Moreau & Gamble, 2020).

Fourth, open materials refer to sharing study components that are necessary for another researcher to conduct a replication of the study, such as survey questionnaires or experimental protocols. For materials to be open, they must be freely and easily accessible to the public. Offering open materials is intended to increase transparency within the research community, and to facilitate replication and extension studies.

Fifth, open access to the products of research includes several components, such as making a preprint of a paper available online (i.e., posting a preprint) for anyone to access or making the published version (i.e., an open access article) of an article freely available online to the public. Both preprints and open access articles are designed to disseminate scientific knowledge to anyone in the world with an internet connection, without having to pay money.

### Reviews of Open Science Practice Use in Scientific Research Articles

Researchers have begun to examine the use of open science practices in peer-reviewed scientific research articles (Adewumi et al., 2021; Hardwicke et al., 2020, 2021; Iqbal et al., 2016; Norris et al., 2021; Wallach et al., 2018). For example, in one study that is comparable to the present study, Hardwicke et al. (2020) focused on a random sample of 250 articles from a diverse collection of social science disciplines published between 2014 and 2017. They observed that 11% of studies included open materials, 7% provided open data, 1% provided open analysis scripts, 1% were replication studies, and surprisingly, none of the 250 studies were pre-registered. They concluded that this lack of transparent and reproducible methods might be undermining the credibility of published scientific research. Another review of 250 articles from psychology, in particular, by Hardwicke et al. (2021) for the same time period reached similar conclusions regarding the use of open science practices, showing that 65% of articles were publicly available (i.e., with no paywall), 14% used open materials, 2% made their data open, and 1% provided open analytic code. Only 5% of the studies were replication studies.

Although these two review articles focused on different disciplines (i.e., social science and psychology, respectively), included fewer articles (i.e., 250 vs. 500 in the present study), and used a slightly older time period as compared to the present study (i.e., 2014–2017 vs. 2016–2019 in the present study), they provide a relevant baseline comparison for the prevalence of open science practices. Gambling studies is a multidisciplinary field that includes psychology and social science-based disciplines, and also uses similar methodologies to these fields, so it would be expected that similar prevalence rates for open science practices might be present in research on gambling-focused topics.

### Open Science in Gambling Studies Research

Few studies to date have discussed open science practices within the field of gambling studies. Notably, four commentaries in gambling studies have examined open science practices and related concepts, as well as provided background on the open science movement and potential paths forward for gambling researchers and key stakeholders who might be new to open science (Blaszczynski & Gainsbury, 2019; LaPlante, 2019; Louderback et al., 2021; Wohl, Tabri, & Zelenski, 2019). These papers address issues ranging from journal policy changes to support open science, to strategies to improve gambling research by avoiding HARKing (*H*ypothesizing *A*fter the *R*esults are *K*nown; Kerr, 1998), to ways to advance open science and replication efforts among gambling researchers, to the protective effects that open science practices might provide for research integrity, even for industry-funded research. Empirical research addressing open science topics within the gambling studies field will provide needed support for some of the tips and strategies these early publications offer. Two such studies currently are available.

First, LaPlante et al., (2021) carried out a survey of gambling research stakeholders to provide a preliminary assessment of opinions on and use of open science practices. Questions of interest asked respondents to report on their engagement with four specific types of open science practices and some reasons for concern about open science (e.g., pre-registration potentially stifling research flexibility or creativity). They determined that minorities of gambling research stakeholders reported either some or extensive experience with open science: 44% indicated extensive or regular experience with open science practices, 31% with pre-registration, 33% with open materials or code, 48% with open data, and 16% with preprinting papers. This study suggested that there remains a broad need for open science education in the gambling studies field. However, the study was preliminary in that it relied upon a small convenience sample.

Second, Heirene et al. (2021) described how well gambling researchers’ study pre-registrations reduce Research Degrees of Freedom (RDoF), or the methodological choices that researchers must make when they collect data, analyze data, and report upon their findings (Wicherts et al., 2016). Their review of 53 available pre-registrations suggested that gambling pre-registrations had low specificity for most of the RDoF that they assessed; that is, the pre-registrations did not sufficiently constrain RDoF. A comparison of pre-registrations with 20 available publications or preprints also revealed that 65% of studies deviated from their pre-registered plans without declaring deviations in the publication or in a Transparent Change document. Heirene et al. (2021), interpreted these findings to suggest that, to date, researchers in the gambling studies field are not taking full advantage of the ability of pre-registration to tighten research practices for the purposes of improving the rigor and replicability of their work.

## The Present Study

The purpose of the present study was to conduct a scoping review of recent gambling literature in order to assess the field’s current engagement with open science practices. The primary research question was: *to what extent do peer-reviewed publications of original (i.e., non-review) quantitative research in the gambling literature use open science research practices, such as pre-registration, open data, and include replication studies with adequate statistical power*? We hypothesized that a minority of peer-reviewed publications in the gambling literature will report using open science research practices or consist of replication studies with adequate statistical power.

## Methods

**Fig. 1:**
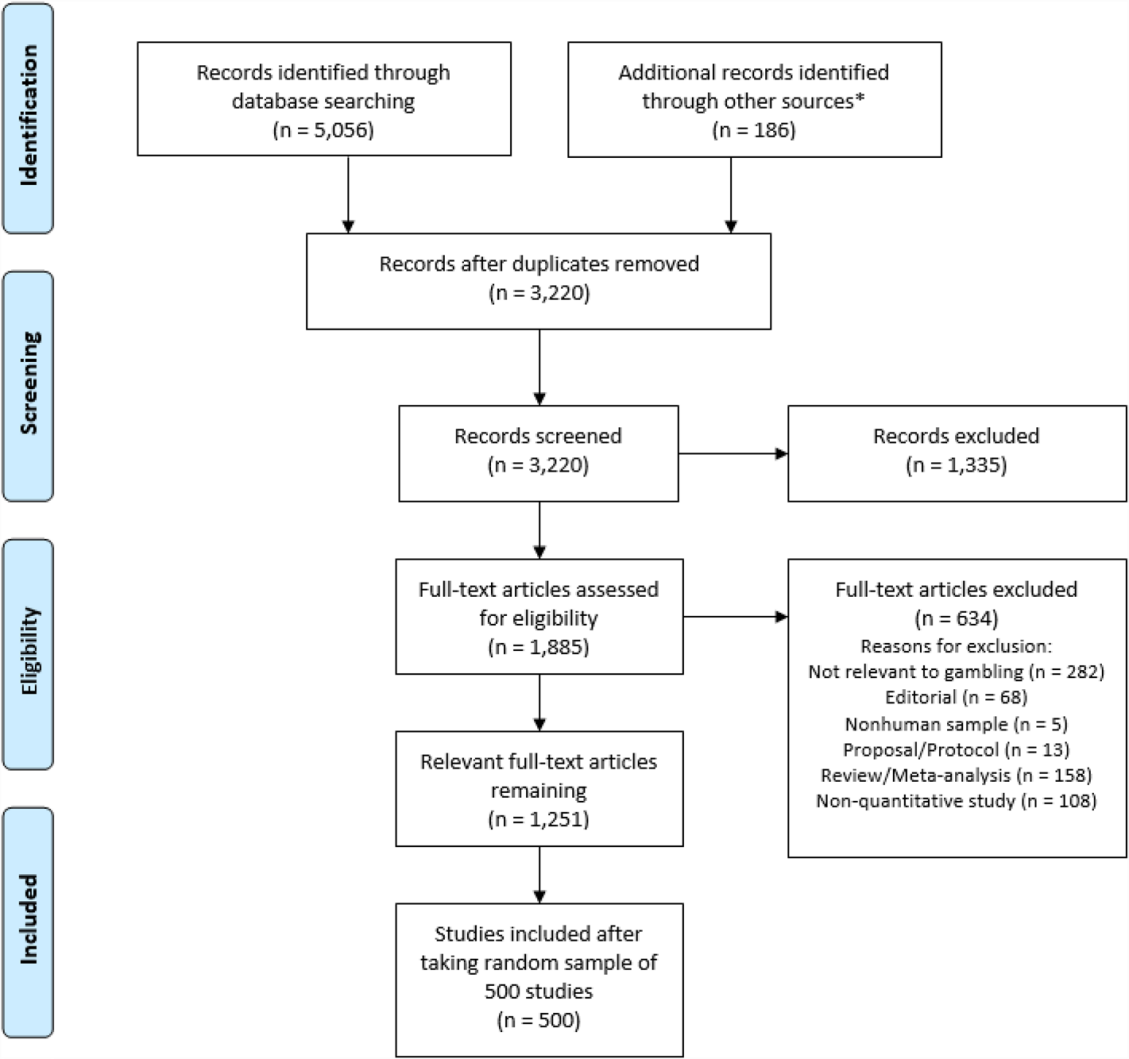
Diagram of study selection process. *We found 186 articles in specialized gambling-focused journals, including the *Journal of Gambling Studies* and *International Gambling Studies*

Our study was pre-registered on the Open Science Framework (OSF) on 9/27/2019 before beginning the study search process (https://osf.io/f2prd), and our data and scripts are available on our OSF project page. All justified amendments to our pre-registered research plan are also included in Transparent Changes documents on our OSF project page. We drafted the protocol using the Preferred Reporting Items for Systematic Reviews and Meta-Analyses (PRISMA) extension for Scoping Reviews (Tricco et al., 2018). We created a PRISMA-style diagram of the study selection and screening process (see Fig. 1).

### Eligibility Criteria

To be included in our scoping review, studies needed to measure or focus on gambling. We defined “gambling” broadly as encompassing the full range of gambling behavior, from gambling without experiencing gambling-related problems (i.e., Level 1), to sub-clinical Gambling Disorder (i.e., Level 2) to Gambling Disorder (i.e., Level 3) (Shaffer et al., 1999). We included studies if they (1) were peer-reviewed, (2) were published between January 1st, 2016 and December 1st, 2019,^2^ (3) were written in English, (4) involved human participants, and (5) described quantitative data analyses. We excluded papers that failed to meet all five inclusion criteria. We also excluded editorials, commentaries, research proposals, study protocols, and review papers, such as meta-analyses, scoping reviews, and literature reviews. We specified the first four inclusion criteria during a database search, and the fifth criteria and the additional exclusion rules during our title, abstract, and full-text inspection.

### Information Sources

To identify potentially relevant studies, we searched the following bibliographic databases covering a variety of scientific disciplines: Medline, Embase (medicine); PsycARTICLES, PsycINFO (psychology); Global Health (public health); the Education Resources Information Center [ERIC] (education); and the Social Science Premium Collection. We supplemented our search by searching the January 1st, 2016 to December 1st, 2019 archives of specialized gambling journals, including the *Journal of Gambling Studies* and *International Gambling Studies*. We exported the research results into EndNote and removed duplicate articles.

### Search Procedures

We selected search terms by reviewing search terms used in published meta-analyses/systematic reviews of gambling. Specifically, we used the following search^3^ terms: “Gambl*”, “betting”, “wager*”, and “ludomania”. We used the appropriate truncation operator and search strategy format for each database, allowing any of the terms (i.e., terms using the Boolean operator OR, as opposed to AND).

### Selection of Sources of Evidence

After we specified our initial sample of studies by employing the first five inclusion criteria during a database search and cross-checking against archives of specialized gambling journals (i.e., Identification), we used Google sheets and Endnote to screen the titles and abstracts of returned studies to assess their relevance (i.e., Screening). We deemed studies to be relevant if they appeared to describe gambling (as opposed to non-gambling-related gaming or video gaming studies, for example, as such articles are sometimes included in the *Journal of Gambling Studies* and *International Gambling Studies*). Vague study titles were retained for full text inspection. We used an iterative process to determine the reliability of our screening process. More specifically, two members of the research team independently screened the titles and abstracts of 10% of all retrieved studies to assess their relevance. They resolved any disagreements through discussion or further adjudication by a third reviewer. Their interrater reliability met our pre-registered criteria of ***κ*** ≥ 0.70 (i.e., Cohen’s ***κ* = **0.790; McHugh, 2012), so they divided the remaining retrieved studies into two groups, and screened their titles and abstracts independently.

Next, three reviewers reviewed full texts of remaining studies to assess study eligibility according to the five inclusion criteria (i.e., Eligibility). They first screened 10% of all remaining full-test studies to assess their relevance. They resolved any disagreements through discussion or further adjudication by a third reviewer. Their interrater reliability for the initial full-text screening met our pre-registered criteria of ***κ*** ≥ 0.70 and **α** ≥ 0.70 (i.e., Fleiss’ ***κ*** = 0.711; and Krippendorff’s **α = **0.711), so they divided the remaining full-text studies into three groups and screened their full-text PDFs independently. This allowed us to confirm that our inclusion criteria were fully satisfied. We only considered studies to be eligible if they meet all inclusion criteria. Eligible studies comprised our baseline sample. From the baseline sample of studies that remained eligible for data charting (i.e., 1,251 studies), we selected a simple random sample without replacement of 500 full-text studies using the *sample()* function in base R for our analytic sample of studies for charting.

### Data Charting Process

We charted data from eligible studies and supplementary sources (e.g., links embedded in publications to pre-registration documents or open data archives) using Google Forms, a secure and customizable online survey platform. We created a custom Google Forms survey (see survey here: https://osf.io/ke3jg/) to record information for this study including all of the data items in Table 1. We used an iterative process to determine the reliability of our charting. Two independent reviewers charted the data items from a randomly selected subset of articles representing 10% of our analytic sample. They resolved any disagreements through discussion or further adjudication by a third reviewer. Prior to resolving disagreements, we assessed the charters’ interrater reliability. We calculated Fleiss’ ***κ*** and Krippendorf’s **α** for each data item. After four rounds of reliability coding, their interrater reliability met our standard for all except six items (see Transparent Change 5 and Transparent Change 6), so we divided the remaining studies into two equal groups and two coders charted them independently. As is described in more detail in our two Transparent Changes documents, to confirm that items that were initially below the reliability threshold were all correct, a Ph.D.-level co-author manually checked all 500 studies.

**Table 1:** Items charted for all studies

Data item	Response format	Response options, if applicable	Information source
Country or countries of origin of sample(s)	Select all that apply	e.g., United States	Publication
Study Funder	Select one	List the funder name(s) or indicate: “Unspecified (no funder listed)” (Funders were then coded by Ph.D.-level co-authors into: Private foundation, Private foundation: indirect industry, University, Industry: direct, Local government, Local government: indirect industry, National government, National government: indirect industry)	Publication
General Past (e.g., 5-year) Author Funding Statement	Select one	Yes, no	Publication
Conflict of Interest Statement	Select one	Conflicts, No Conflicts, No Conflict of Interest Statement	Publication
Month and year of study publication	Fill-in-the-blank	e.g., March, 2016	Publication
Power analysis reported	Select one	Yes, *a priori*, Yes,* post-hoc*, no	Publication
Sample size(s) (at baseline, if applicable)	Fill-in-the-blank	e.g., *N* = 300	Publication
Specific sample description	Fill-in-the-blank	e.g., “gambling treatment clients recruited from 2 treatment sites”	Publication
Study design	Gated question, Select one	(1) Experimental or Observational (2a) Experimental: Randomized controlled trial, Non-randomized trial (2b) Observational: Cross-sectional, Prospective Cohort, Retrospective Cohort, Case–control, Case Series/Case Study	Publication
Registration status	Select one	Pre-registration available, Pre-registration and registered report available, Pre-registration not available, No Pre-registration	Publication, with verification that pre-registration is accessible online
Open data	Select one	Yes, no	Publication, with verification that data are accessible
Open notebook	Select one	Yes, no	Publication, with verification that notebook is accessible
Open materials (i.e., study components, such as study instructions, surveys, and other aspects, needed to reproduce)	Select one	Yes, no	Publication, with verification that materials are accessible
Open analytic code	Select one	Yes, no	Publication, with verification that code is accessible
Open access	Select one	Yes, no	Availability through Google Scholar, Open Access Button (openaccessbutton.org) or open access on journal website
Preprint	Select one	Yes, no	Availability through Google Scholar, OSF or PsyArXiv
Replication status	Select one	Original study, conceptual replication, primary replication	Publication
Gambling concept(s) measures	Select one/Fill-in-the-blank	Gambling participation/involvement, Presence/severity of gambling problems, Other gambling concept(s) (specify)	Publication
Major finding(s)	Fill-in-the-blank (narrative summary; 2–3 sentences)		Publication

### Data Items

We charted the studies on the data items listed in Table 1. Data items derive, in part, from Transparency and Openness Promotion criteria (TOP; Center for Open Science, 2019) and two recent scoping studies of open science research practices that were publicly pre-registered before we developed the pre-registration for the present study (Adewumi et al., 2021; Hardwicke et al., 2020). During charting, we modified the specific guidelines for assessing a few of the data items in Table 1.^4^ For our charting purposes, we charted ‘yes’ for open access if and only if the final version of the study was available for download on the official journal website without logging in or paying money. We charted ‘yes’ for preprint availability if the preprint was a version of the article that was publicly available for download, yet was not typeset by the journal itself in its published version.

### Data Analysis and Synthesis of Results

All analyses were conducted in R version 3.6.2 (R Core Team, 2019). For our main synthesis, we report tables of counts and percentages, with 95% confidence intervals, related to our data items (e.g., the count of and percentage of all studies that were open access). Using year of publication, we provide a year-by-year summary count of the number and percentage of publications per year that used any single open science research practice from our data items.

To better understand how study design might be related to open science practices, we analyzed ten cross-tabulations of study design (i.e., experimental vs. observational; in the columns) and ten study characteristics related to open science (in the rows). We also investigated how open science practices might have increased, decreased, or remained stable over time by analyzing ten cross-tabulations of year of study publication (in the columns) and these same ten study characteristics (in the rows). We tested for the significance of any associations in our cross-tabulations with Chi-square tests using a *p* < 0.05 criteria for statistical significance and Cramer’s *V* to interpret the size of effects.

Using the final charted data, we created a master spreadsheet with a Characteristics of Included Studies (COIS) table that summarizes study-level information for all data items (https://osf.io/r2n3m/). We generated a unique numeric identifier for each study (i.e., a COIS ID) to identify studies. In the Results section, we synthesize the outcomes of our review narratively to provide an overall description of the extent to which open science practices are used in the body of relevant research evidence available. The R scripts used to conduct the reliability analyses, random study selection, and analyses described in the Results are available on our OSF Project page (https://osf.io/xw7gf/).

## Results

### Open Science-Related Measures

Table 2 displays counts, percentages, and 95% confidence intervals for open science practices in our sample. Rates of engagement with open science practices ranged from 0% for open notebooks to 35.2% (*n* = 176) for open access. Overall, 54.6% (*n* = 273) of studies used *at least* one of the nine open science practices. Of the 500 studies, we found that 4.4% (*n* = 22) reported conducting an *a priori* power analysis and 2.0% (*n* = 10) reported conducting a *post-hoc* power analysis. For replication status, 488 (97.6%) studies were original studies and 12 (2.4%) were conceptual replication studies. None of the 500 studies were primary (i.e., direct) replications. Thus, our research hypothesis stating that a minority of studies will use open science practices is partially supported.

**Table 2:** Percentages, counts and 95% confidence intervals for open science items

Charted item	%	Count	95% CI
Any open science practice	54.6	273	(50.22 – 58.91)
Pre-registration	1.6	8	(0.81 – 3.13)
Open data	3.2	16	(1.98 – 5.13)
Open notebook	0	0	(0 – 0)
Open access	35.2	176	(31.14 – 39.48)
Open materials	7.8	39	(5.76 – 10.49)
Open code	1.4	7	(0.68 – 2.86)
Preprint	15.0	75	(12.14 – 18.40)
Power analysis	6.4	32	(4.57 – 8.90)
Replication study	2.4	12	(1.38 – 4.15)

*N* = 500; Note: Confidence intervals were calculated for each percentage based on the normal distribution assumption and used Wilson’s score method as described in Newcombe (1998)

### Past Funding Statement and Conflict of Interest Statements

As shown in Table 1, we also coded the presence of past funding (e.g., In the past 5 years, author 1 has received funding from X, Y, Z, etc.) and conflict of interest statements. Such statements provide transparency about potential and actual conflicts of interests, and are intended to prevent obscuring funding sources for each author. For past funding statements, 13.2% (*n* = 66) of studies included this statement for at least one author. For conflict of interest statements, 21.4% (*n* = 107) of studies included a statement with potential conflict(s), whereas 56.4% (*n* = 282) included a statement that indicated no conflicts existed. Thus, overall, 77.8% (*n* = 389) of studies included a conflict of interest statement.

### Sample Size and Study Design

Sample sizes for studies varied considerably, ranging from *n* = 1 to *n* = 267,367, with a median sample size of *n* = 328 and mean sample size of *n* = 3,321 (SD = 16,997).^5^ For study design, there were substantially more observational studies (82.0%, *n* = 410) than experimental studies (18.0%, *n* = 90).

### Gambling Concept(s) Measured

For all studies, we coded whether a study measured: gambling participation/involvement, the presence/severity of gambling problems, and/or other gambling concept(s) (specify). We found that 84.6% (*n* = 423) measured gambling participation/involvement, 49.0% (*n* = 245) measured presence/severity of gambling problems, and 36.6% (*n* = 183) measured *both* gambling participation/involvement and presence/severity of gambling problems. For other gambling concepts, 33.6% (*n* = 168) of studies measured these, and these concepts included a wide variety of different concepts, ranging from gambling-related cognitions to parental gambling to gambling motives.

#### Planned Confirmatory Analyses

##### Study Design and Open Science Practice Variables

Table 3 shows the comparisons of frequencies for open science practice variables for experimental studies and observational studies. A significantly greater percentage of experimental studies used at least one open science practice, as well as pre-registration and a power analysis. There were no significant differences between experimental and observational studies for the other seven items.

**Table 3:** Cross-tabulation and Chi-square tests of study design and open science practices

Item charted	Experimental (*n* = 90)	Observational (*n* = 410)	**χ**^**2**^	*p*-value	Cramer’s *V*
	% (Count)	% (Count)			
Any open science practice	65.6 (59)	52.2 (214)	4.7890	0.0286	0.103
Pre-registration	6.7 (6)	0.5 (2)	14.1867	0.0002	0.189
Open data	4.4 (4)	2.9 (12)	0.1682	0.6818	–
Open notebook	0 (0)	0 (0)	–	–	–
Open access	37.8 (34)	34.6 (142)	0.1968	0.6573	–
Open materials	6.7 (6)	8.0 (33)	0.0509	0.8214	–
Open code	2.2 (2)	1.2 (5)	0.0565	0.8120	–
Preprint	17.8 (16)	14.4 (59)	0.4251	0.5144	–
Power analysis is reported	15.6 (14)	4.4 (18)	13.5509	0.0002	0.175
Replication study	2.2 (2)	2.4 (10)	0.00	1.0000	–

Percentages shown were calculated separately for experimental and observational study designs. Cramer’s *V* effect size was only reported for statistically significant differences and is equivalent to **φ** in 2 × 2 tables. All Chi-square tests were based on 1 degree of freedom

##### Year of Study Publication and Open Science Practice Variables

Table 4 shows the comparisons of open science practices over time. The number of studies included from each year was relatively similar. None of the open science practice variables yielded significant differences across the four-year period.

**Table 4:** Cross-tabulation and Chi-square tests of year of publication and open science practices

Item Charted	2016 (*n* = 115)	2017 (*n* = 124)	2018 (*n* = 132)	2019 (*n* = 129)	**χ**^**2**^	*p*-value	Cramer’s *V*
	% (Count)	% (Count)	% (Count)	% (Count)			
Any OS practice	49.6 (57)	57.3 (71)	**60.6 (80)**	50.4 (65)	4.3738	0.2238	–
Pre-registration	0 (0)	**2.4 (3)**	2.3 (3)	1.6 (2)	2.7801	0.4268	–
Open data	0.9 (1)	**4.8 (6)**	4.5 (6)	2.3 (3)	4.1811	0.2426	–
Open notebook	0 (0)	0 (0)	0 (0)	0 (0)	–	–	–
Open access	34.8 (40)	37.1 (46)	**40.2 (53)**	28.7 (37)	4.0258	0.2587	–
Open materials	3.5 (4)	**11.3 (14)**	8.3 (11)	7.8 (10)	5.1398	0.1618	–
Open code	0 (0)	**2.4 (3)**	2.3 (3)	0.8 (1)	3.6594	0.3007	–
Preprint	13.0 (15)	16.1 (20)	14.4 (19)	**16.3 (21)**	0.6728	0.8796	–
Power analysis is reported	2.6 (3)	5.6 (7)	8.3 (11)	**8.5 (11)**	4.6754	0.1972	–
Replication study	1.7 (2)	**3.2 (4)**	2.3 (3)	2.3 (3)	0.5876	0.8993	–

Percentages shown were calculated separately for each year. All Chi-square tests were based on 3 degrees of freedom. **Emboldened** counts and percentages represent the highest values over the four years for each item

*OS* open science

#### Unplanned Exploratory Analyses

##### Evidence Map of Open Science Practices, Gambling Concept(s) Measured and Study Design

During the course of the study, we conducted several sets of unplanned exploratory analyses, which we report in this section. First, similar to the evidence map created in Gray et al.’s (2021) scoping review of gambling and self-harm, we created an evidence map (see Supplemental Table 1 in Online Supplement) that shows the COIS ID numbers for studies based on the gambling concept(s) they measured, whether they used each open science practice, and study design (i.e., experimental **in bold** and observational un-bolded). This table indicates that studies focused on gambling participation the most, followed by presence or severity of gambling problems, and finally, other gambling concepts. The most common open science practice was open access, followed by preprint, and open materials. Interestingly, while very few studies were pre-registered, the majority of studies that did pre-register (75%) were experimental.

##### Funder Type and Open Science Practices

In a second set of exploratory analyses, to better understand how studies were funded and how this might have been associated with open science practice use, we collapsed the nine funder categories into three conceptual groupings for analysis, including Group 1: Private foundation, University or No Funding, Group 2: Government, and Group 3: Industry Direct. These unplanned exploratory analyses were developed after conducting the confirmatory analyses. We selected a three-group approach to maintain sufficient statistical power and expected cell counts for conducting Chi-square analyses. Table 5 shows these analyses. Direct industry-funded studies were more likely to use any open science practice, open access, and open materials. Industry-funded studies were also more likely to report the use of a power analysis.

**Table 5:** Unplanned exploratory analysis of study funder type and open science practices

Item Charted	Private foundation, University or No Funding (*n* = 257)	Government (*n* = 213)	Industry Direct (*n* = 30)	**χ**^**2**^	*p*-value	Cramer’s *V*
	% (Count)	% (Count)	% (Count)			
Any open science practice	48.6 (125)	59.6 (127)	70.0 (21)	8.7245	0.0127	0.132
Pre-registration	0.8 (2)	2.3 (5)	3.3 (1)	2.4307	0.2966	–
Open data	1.9 (5)	4.7 (10)	3.3 (1)	2.8439	0.2412	–
Open notebook	0 (0)	0 (0)	0 (0)	–	–	–
Open access	29.2 (75)	40.4 (86)	50.0 (15)	9.4616	0.0088	0.138
Open materials	5.4 (14)	9.4 (20)	16.7 (5)	6.0058	0.0496	0.110
Open code	1.2 (3)	1.9 (4)	0.0 (0)	0.8792	0.6443	–
Preprint	14.8 (38)	16.0 (34)	10.0 (3)	0.7522	0.6865	–
Power analysis is reported	7.4 (19)	3.8 (8)	16.7 (5)	8.1876	0.0167	0.128
Replication study	1.9 (5)	2.8 (6)	3.3 (1)	0.4962	0.7803	–

Percentages shown were calculated separately for each funder grouping. All Chi-square tests were based on 2 degrees of freedom

##### Study Citation Count and Open Science Practices Used

In a third set of exploratory analyses (see Table 6), we examined the number of citations that each article received by conducting a Google Scholar search for each article title during the week of August 23rd, 2021. Next, to examine how open science practices might have been associated with citation counts, we ran independent sample *t*-tests comparing the mean citation count for studies that did or did not use any open science practice, as well as each of the nine separate open science practices. These results showed that studies that used any open science practice (*t* = −2.1423;* p* < 0.05; *d* = 0.186) and those that had open access availability (*t* = −2.0727; *p* < 0.05; *d* = 0.218) both had higher mean citation counts. We found no other significant differences for any of the eight remaining open science practices. In response to reviewer comments, we also conducted unplanned exploratory analyses of any open science practice and the nine specific open science practices, and citation counts, separately within observational studies (*n* = 410) and experimental studies (*n* = 90). These results are reported in Supplemental Tables 2 and 3 in the Online Supplement. We did not find any significant differences in these analyses.

**Table 6:** Unplanned exploratory analysis of citation counts and open science practices

	Citation count		*t*	*p-*value	Cohen’s *d*
	Did not use practice *M* (SD) Median	Used practice *M* (SD) Median			
Any open science practice	15.2 (16.5) 11.0	19.0 (23.7) 12.0	− 2.1423	0.0327	0.186
Pre-registration	17.2 (20.9) 12.0	19.1 (18.5) 13.0	−0.2850	0.7836	–
Open data	16.9 (18.8) 11.0	28.7 (53.8) 15.0	−0.8752	0.3952	–
Open notebook	–	–	–	–	–
Open access	15.7 (17.2) 11.0	20.2 (26.1) 13.0	−2.0727	0.0392	0.218
Open materials	16.5 (17.7) 11.0	26.5 (42.5) 13.0	−1.4588	0.1526	–
Open code	16.8 (18.7) 11.0	49.1 (79.0) 17.0	−1.0824	0.3206	–
Preprint	16.4 (17.5) 11.0	22.4 (33.9) 13.0	−1.4935	0.1392	–
Power analysis is reported	17.3 (21.1) 11.5	17.0 (16.0) 13.0	0.0872	0.9310	–
Replication study	17.3 (21.0) 11.0	18.2 (14.9) 13.0	0.2075	0.8391	–

Table shows means (*M*), standard deviations (SD) and medians for any open science practice and each of the nine open science practices separately. All *t*-tests used Welch’s adjustment for unequal variances

## Discussion

The scientific study of open science practices in the gambling research field is limited. This scoping review examined the use of open science practices in a random sample of 500 gambling studies research publications for 2016 through 2019. More than half (54.6%) of the studies used at least one open science practice. Although this is not a large majority, it ran counter to our expectations that a minority would have used at least one open science practice. We also found that—with the exception of open access and preprint posting—all open science practices had very low prevalence (i.e., fewer than 10% of studies used each practice). Temporal trends might be suggestive of increasing open science practice use over time, but they were not statistically significant. Small cell counts and limited statistical power likely prevented us from observing statistically significant changes for each open science practice by year.

We found that experimental studies were more likely to have been pre-registered and to report a power analysis. This suggests that experimental studies—which often have small sample sizes and registration requirements for participant recruiting (e.g., in clinical trials)—might be more concerned with the necessary sample size to ensure adequate statistical power and must also pre-register to comply with mandates (e.g., for FDA-funded trials). In contrast, observational studies often (but not always) have larger sample sizes, even into the 1,000s. For these types of studies, statistical power might be less of a concern and no regulatory mandates exist to encourage pre-registration.

Unplanned exploratory analyses of study funder groupings showed that industry funded studies tended to use more open science practices, including open access, open materials, and a power analysis, when compared to government, private foundation, university funded, or unfunded studies. There are two potential explanations for this pattern of findings. First, it is possible that researchers—being aware of criticisms of industry funded research and thus seeking objectivity (see Cottler et al., 2016)—intentionally use more open science practices to help emphasize the firewall of independence between researchers and their industry funders. Indeed, this particular issue is discussed by Louderback et al. (2021), going so far as to develop guidelines for the integration of open science practices within industry funding models. A second, and more mundane explanation, which is relevant for open access in particular, is the desire for industry funders to view and distribute the products of research without paywalls. The findings that government-funded studies also were more likely to be open access might also reflect this explanation; government funders are increasingly mandating that published study manuscripts be made available with open access status (Rabesandratana, 2019), which undoubtedly impacts whether studies are indeed publicly available without paywalls.

Additional unplanned exploratory analyses of citation counts showed that studies that used any open science practice or that had open access availability were cited with greater frequency. It is possible that using more open science practices made studies more visible to larger audiences, for example, by allowing interested readers to download studies without a paywall, thus increasing citation counts. These findings indicating higher citation rates among open access articles are consistent with studies in other scientific disciplines (Basson et al., 2021; Kousha & Abdoli, 2010; Norris et al., 2008). Further, a recent systematic review (Langham-Putrow et al., 2021) noted a small citation advantage for open access articles. Research suggests that a citation advantage for open access articles is likely due more to greater article visibility and accessibility, rather than the self-selection of more rigorous or impactful articles into being open access (Gargouri et al., 2010; Langham-Putrow et al., 2021). However, we were unable to empirically test this possibility in the present study. Unlike Colavizza et al. (2020), however, we did not find that studies with open data received more citations. The low prevalence and small cell counts for other practices might have prevented us from finding similar citation rate patterns for open data, code, and materials. Low prevalence and small cell counts might have also played a role in the non-significant results for citation counts in exploratory analyses within observational and experimental studies separately. Follow up studies should replicate these analyses when open science practices have developed to a greater degree and are more prevalent in gambling studies.

Importantly, the present study represents the first scientific scoping review of open science practice usage in contemporary gambling studies research. We reviewed the largest representative sample of publications to date in reviews of open science practices, which provides more precise point estimates (i.e., narrower confidence intervals). Our results showing that open science practices are limited in published gambling research studies are largely consistent with other recent scoping reviews of open science practice use in the social sciences (Hardwicke et al., 2020, 2021) and with gambling researchers’ self-reported use of open science practices (LaPlante et al., 2021); this key finding suggests that there is considerable progress needed to be made in terms of integrating open science principles into gambling studies research.

It also raises potential concerns about limited methodological transparency and essential materials (e.g., data, analysis scripts, etc.) sharing in gambling studies research, both of which can potentially result in limitations in the rigor, relevance, and replicability of scientific research (Allen & Mehler, 2019; Gorgolewski & Poldrack, 2016). A lack of sharing study materials might also act as a barrier to scientific innovation (Conrado et al., 2017), and thus could slow efforts to build a body of knowledge that leads to the implementation of evidence-based approaches to the identification, prevention and treatment of Gambling Disorder and gambling-related harm. The next sections provide a more in-depth discussion of these implications and include practical suggestions for enhancing the uptake and use of open science practices among gambling researchers. These suggestions can also be extended to all scientific disciplines.

### Practical Implications

The present findings suggest a need for more researchers to be involved with open science within the multidisciplinary field of gambling research. One effective source of change can be young researchers, such as students and those who are early on in their scientific career. If we can teach engagement with open science knowledge and practices early on, it can become a regular and habitual part of the research process as young researchers move forward and advance in their careers (see Allen & Mehler, 2019 for open science tips for early career scholars). Several resources exist to help provide information and entry points for making these practices a part of research. For example, Banks et al. (2019) provided clear answers to 18 questions about open science practices. These questions and answers provide information on the principles of open science, the benefits and challenges of engaging in open science practices, the progress that has been made thus far to adopt open science practices, and steps that could to be taken in order to facilitate the uptake of open science practices. This article is one of many resources (also see Gehlbach & Robinson, 2021) that clearly break down the principles of open science and detail how researchers can take steps to engage in such practices.

Organizations and educational programs have already been working to teach young researchers about open science, including the Center for Open Science, who run the Open Science Framework, and the FOSTER Open Science organization (FOSTER, 2021). FOSTER is an organization based in the European Union (E.U.) that aims to create a cultural change in which open science is integrated into research practices and rewarded within the research community. The organization hopes to reach this goal through a fully open and free training program that provides a continued support network once the training is completed. Individuals who complete the training receive a badge as an Open Science Ambassador. FOSTER also created resources such as the Open Science Toolkit and the Open Science Training handbook, providing additional opportunities to promote and take advantage of this program to disseminate information about the benefits of open science and how it can be integrated into research practices.

In order to engage more established researchers in utilizing open science practices, academic institutions might consider enacting requirements for these practices as conditions for tenure and promotion. These requirements might be similar to requirements that already exist for evidence of the number of publications or of citations of one’s work in order to progress within the institution (Mu & Hatch, 2021). Additionally, integrating open science practices as a basic standard for research methods in undergraduate, graduate, and continuing education courses would help to influence an overall culture shift towards embracing such principles and practices.

### Policy Implications

Because our findings suggest that there is a need for increased engagement with open science practices, policies might be considered to help facilitate knowledge and engagement. As the gatekeepers to publications, journals have the ability—and some might argue the responsibility—to take a leading role in this transition by creating policies that promote open science practice use in published peer-reviewed research (see Nutu et al., 2019 for initial steps taken by psychology journals). In a recent article, Aguinis and colleagues (2020) discussed recommendations for scientific research to narrow the gap between open science theory and practice. Specifically, they provide 10 actionable recommendations of policy changes for authors and journals that could help narrow the science-practice gap in open science. An example of one recommendation is for Editors to introduce a policy for results-blind review, which involves submitting a full manuscript for review that excludes the results section. This practice might reduce Editor and reviewers’ positive results bias, and promote a stronger focus on the theoretical and practical impact of the study itself rather than whether or not the findings were statistically significant (see Ingre & Nilsonne, 2018). Other important recommendations for journals discussed by Aguinis et al. (2020) include: (1) requiring a pre-registration for every study, (2) publicly sharing the data, code, and materials for all studies, (3) creating a review track that includes Registered Reports, (4) providing an online archive for each journal article, such as the Open Science Framework, for authors to post their study materials, (5) creating a best-paper award that is based on the use of open science criteria, and (6) providing access to open science training for all research stakeholders.

Beyond these suggestions for journals in particular, recommending that funding agencies modify their policies in order to facilitate open science practices is another potential avenue for change. For example, funding agencies could require that authors receiving funding must pre-register and conduct a power analysis in their studies, and as noted above, government funding bodies in the United States (U.S.) and E.U. have already started mandating that peer-reviewed publications be available via open access (Rabesandratana, 2019). Moreover, funders could also encourage that de-identified data collected for a study be made public after a period of time, thus facilitating open data sharing. All of these recommendations for policy changes require minimal additional resources besides time investment. However, both funders and research institutions must recognize and account for this additional time investment to enable researchers to engage with open science practices.

### Study Limitations

Our study is not without limitations. First, we only included 500 articles out of the total sample (*N* = 1251) of relevant articles that we found during our database search. However, the articles were randomly selected from the total sample so they are representative of all of the articles that met the inclusion criteria. Nonetheless, we reported all of our point estimates with confidence intervals to model potential sampling error. Second, we only included articles that were published between January 1, 2016 and December 1, 2019. As open science practices have been on the rise in recent years, it is likely that we would have found even less engagement with open science practices in the gambling literature if we had included studies from before 2016. In addition, the COVID-19 pandemic would have confounded practices used in studies published in 2020 and 2021. Third, we charted items using an agreed upon coding scheme to maintain consistency, and other authors might have developed a different coding scheme for charting data items. For example, we coded conflict of interest statements as being present for a study based on statements with different headers (e.g., “Competing Interests Statement”, “Declaration of Competing Interests”, “Conflict of Interest Statement”), and other researchers might have only coded a study as having a conflict of interest statement if it was titled “Conflict of Interest Statement”.

### Directions for Future Research

Based on our study, there are five avenues for future research. First, when collecting data on the articles reviewed in this study, we also recorded the titles of the journals that these articles were published in. Future studies should look into exploring and comparing open science practices across various journals. There is potential for an association between journals and their preferred open science practices, or a pattern showing that publications in some journals practice open science to a greater degree. The same approach could be extended to compare the presence of replication studies across journals. To our knowledge, no scoping reviews comparing open science and replication practices across specific journals or journal groupings (e.g., by impact factor and/or fully open access vs. non-fully open access) have been conducted.

Second, it would be useful and interesting to test the extent to which studies that used more open science practices were more likely to report non-significant findings (i.e., null results). As we described previously, one of the key motivations for the open science movement was to increase transparency in the research process and to prevent researchers from engaging in practices such as “*p*-hacking” (Berman et al., 2018) or HARKing (Bosco et al., 2016; Kerr, 1998) in order to be able to report significant findings that many journals prioritize over null findings. It is therefore reasonable to assume that when authors engage in more open science practices (e.g., pre-registration), they are more likely to report findings that are not significant. Existing evidence has found that Registered Reports in psychology do tend to yield far lower rates of statistical significance as compared to regular articles (Scheel et al., 2021), so examining this relationship in other disciplines would be an important contribution in this area. Beyond Registered Reports, it would be interesting to examine the association between other open science practice use and reporting non-significant findings, and to see whether there are other moderating factors that play a role in this relationship, such as the journal an article was published in and/or the funder(s).

Third, another fruitful avenue of research is related to ways that the scientific community can promote the use of open science practices for gambling researchers through educational programs (Crüwell et al., 2019) and journal incentives (e.g., Open Science Badges; see Grahe, 2014). Given the findings in the present study and other research which found that only a minority of gambling research stakeholders report engaging in open science practices (LaPlante et al., 2021), additional research into motivations for and barriers to the use of open science practices is warranted, specifically for researchers publishing gambling literature and for science more broadly. Because we now know that the use of open science practices was relatively low in general for recent gambling-focused research, a better understanding of attitudes towards open science will help provide next steps for such practices in the months and years to come.

Fourth, future research could look into the standardization of conflict of interest statements and recent funding statements across articles, and whether the lack of clear statements regarding funding is associated with industry-funded or other types of funding arrangements for research. Our data charting process showed that there is not currently a standard for the conflict of interest statement and statements listing funding that a researcher has received in the past. It would be interesting to see if the articles with certain types of statements or those without such statements altogether were associated with certain funding sources, and in turn if those studies had more significant (or non-significant) findings. Creating clear guidelines (e.g., *International Gambling Studies’* five-year past funding disclosure policy) across journals will require authors to be transparent about their funders and their research more generally.

Fifth, given the rise in open science practices and the current peer-review process, it is important that future research examines the extent to which reviewers are educated and informed about open science, including pre-registration and deviations from pre-registered plans (see Heirene et al., 2021), separation of confirmation and exploratory analyses, and scientific concerns (e.g., HARKing). The current peer-review process can include reviewers suggesting changes to the methodology, data analyses, hypotheses and presentation of results (Jana, 2019), which can potentially lead to questionable research practices including HARKing, selective outcome reporting (e.g., asking to not report non-significant results), or *p-*hacking in revised study manuscripts, potentially leading to publication bias due to the omission or removal of non-significant findings. In response, future studies could investigate how often reviewers suggest deviations from pre-registered study plans and how differences in levels of open science knowledge across reviewers might impact elements of publication decisions.

### Conclusion

Our findings suggest a large potential for growth in open science practice usage among gambling studies researchers, which also signals a substantial need for education on open science topics for established and aspiring researchers alike. Although the prospect of integrating open science as a standard methodological practice might be daunting to some, it is clear that the benefits of this research evolution will be multifold. However, alongside efforts that promote structural changes in how researchers approach their work must be efforts to promote structural changes to the work environment that support open science practices. Research institutions, journals, and funders must support researchers to engage with open science practices by providing sufficient time, training, and incentives (e.g., publication/promotion requirements) in order to maximize the uptake of open science practices and, in doing so, substantially improve the quality and transparency of the scientific evidence base.

## Supplementary Information

Below is the link to the electronic supplementary material.

Supplementary file1 (DOCX 26 kb)

## Data Availability

Data and materials for this article are available on the Open Science Framework (https://osf.io/xw7gf/).

## Footnotes

1 We refer to “gambling studies” as a multidisciplinary research field that examines micro-level (e.g., gambling frequency) and macro-level (e.g., national prevalence of gambling) gambling behavior, problem gambling, DSM-5 criteria Gambling Disorder, and risk factors for experiencing gambling-related harm.

2 This time period is particularly relevant for open science because the Pre-registration Challenge—which offered researchers $1,000 for each successfully published pre-registered study—was originally launched by the Open Science Framework in December 2015, and led to greater interest in open science and a substantial rise in pre-registered research (Mellor et al., 2019). The end date for the search was selected because we originally conducted the database search in December, 2019, which was only 2 to 3 months prior to the rise of the COVID-19 pandemic, which has greatly impacted open science and science more generally worldwide.

3 Our initial pre-registered search terms included “gaming”; however, this search strategy yielded many irrelevant studies (e.g., examining video gaming or sports games), so we transparently detailed this modification in a publicly posted Transparent Change document on OSF (i.e., Transparent Change 2).

4 During the charting process, the team made changes to the guidelines for assessing a few of the data items. These items included the study date, open access, preprint, and, to a much greater extent, the study funder. The modifications were made to increase the consistency and clarity of the charting process for coders. These changes are described in full detail in Transparent Change 1 and Transparent Change 4, which are publicly available on OSF.

5 While not extremely large, especially for the median, the median and mean sample sizes do exceed the long-standing conventional recommendations for minimum sample sizes (i.e., *n* >  ~ 50; see Green, 1991) for conducting many parametric statistical tests (e.g., regression). However, depending on the desired effect size (i.e., small, medium or large), alpha level (e.g., α = 0.05) and power (1–β), these sample sizes might be insufficiently powered to detect actual statistically significant results (Cohen, 1992; O'Keefe, 2007), yielding a greater probability of incurring a Type II error (i.e., the null hypothesis that there is no difference or effect is incorrectly accepted as true, preventing the detection of an actual statistically significant difference or effect that would provide support for the alternative hypothesis).
