# Supplementary material for: Open Science Practices in Gambling Research Publications (2016–2019): A Scoping Review
Source: J Gambl Stud. 2022 Jun 9;39(2):987–1011. doi: 10.1007/s10899-022-10120-y (PMC9178323; doi:10.1007/s10899-022-10120-y)
Supplement: Supplementary file 1 — Supplementary file1 (DOCX 26 kb) [file 10899_2022_10120_MOESM1_ESM.docx]

**Online Supplement for:**

**Open Science Practices in Gambling Research Publications (2016 – 2019):**

**A Scoping Review**

Supplemental table 1: Evidence map using COIS study IDs

| *Open Science Practice Included* | *Gambling participation/involvement (e.g. frequency, money spent, type of gambling activity, etc.)* | *Presence/severity of problem gambling (e.g., problem gambling screen status, gambling-related harm, etc.)* | *Other gambling concepts (e.g., family gambling exposure, gambling cognitions, etc.)* |
| --- | --- | --- | --- |
| Pre-registration | ***10****,* ***16****,* ***304****,* ***376****,* ***422*** | ***10****,* ***16****, 276, 451* | ***233*** |
| Open data | *34, 126, 174, 189,* ***285****, 357, 375, 428, 440,* ***449****, 453, 464,* ***465*** | *34, 74, 174, 189, 318, 375,* ***417****,* ***465*** | *74, 126, 375, 464* |
| Open notebook | *_* | *_* | *_* |
| Open access | *4, 5, 6, 8, 9,* ***10****, 11, 13, 24, 32, 34, 35, 37,* ***44****, 48,* ***50****, 55,* ***82****, 97, 116, 120, 122, 123, 137, 138, 144, 153, 173, 178, 188, 189, 192,* ***198****, 202, 217, 251,* ***262****, 271, 275,* ***281****, 296,* ***302****,* ***303****,* ***304****, 307, 308,* ***310****, 314, 339, 346, 351, 352, 353, 354, 355, 356, 357,* ***358****, 359, 360, 361,* ***363****,* ***365****, 366, 368,* ***369****, 370, 373, 375,* ***376****,* ***377****, 379, 381, 382, 383, 385, 387, 388, 389, 390, 393, 395, 396, 399, 400, 401,* ***402****, 403, 404, 406, 409, 411, 412,* ***413****, 414,* ***415****, 416, 419, 420,* ***422****, 423, 424, 425, 426, 427, 428, 430, 431, 433, 434, 435, 437, 440, 442,* ***445****,* ***446****, 448,* ***449****, 450, 452, 453, 454,* ***456****, 464, 466, 469, 471, 474, 475, 476, 477, 480,* ***481****, 483, 486, 487,* ***488****,* ***489****, 490,* ***491****, 492, 498, 500* | *4, 5, 7, 8, 9,* ***10****, 11, 13, 24, 34, 37,* ***44****, 48, 73,* ***82****, 137, 150, 154, 181, 188, 189, 191, 192, 202, 237, 271, 272, 296, 299,* ***303****,* ***310****, 318, 339, 346, 353, 354, 359, 361, 367, 368,* ***369****, 370, 375, 379, 380, 381, 382, 384, 387, 388, 390, 391, 398, 399, 400, 401,* ***402****, 406, 411, 412, 414,* ***415****,* ***417****, 423, 427, 430, 431, 434, 435, 436, 443, 444,* ***445****,* ***446****, 447, 451, 455,* ***458****,* ***459****, 466, 467, 468, 471, 472, 473, 475, 476, 477, 478,* ***481****, 482, 486, 487,* ***488****, 497, 500* | *11, 35,* ***44****, 48,* ***69****, 97, 137, 138, 178, 181, 202,* ***262****, 275,* ***284****, 296, 307, 308, 346, 352, 355, 368,* ***369****, 375, 379, 388, 389, 396, 401,* ***402****, 403, 409, 412,* ***415****, 424, 427, 430, 431, 433, 443, 444,* ***446****, 450, 454, 464, 467, 468, 469, 472, 476, 480,* ***488****,* ***489****,* ***491*** |
| Open materials | *34, 47, 48,* ***72****, 99, 126, 138, 146, 174, 187, 189, 196, 244, 283, 295, 296, 298, 352, 353, 357, 364, 366, 375, 401, 428, 430,* ***445****,* ***446****, 453, 464,* ***465****, 475,* ***491****, 493* | *34, 48, 99, 145, 146, 174, 189, 213, 244, 268, 283, 296, 353, 375, 401,* ***417****, 430,* ***445****,* ***446****,* ***465****, 468, 475* | *47, 48, 99, 126, 138, 213, 244, 295, 296, 352, 375, 401, 430,* ***446****, 464, 468,* ***491****, 493* |
| Open code | *174, 189, 357, 428, 453,* ***465*** | *174, 189,* ***417****,* ***465*** | *_* |
| Preprint | ***44****, 47, 48, 49, 53, 54, 57, 63, 65,* ***87****, 108, 109, 117,* ***119****, 147,* ***162****, 172, 174, 176, 189, 203, 205, 210, 220, 221, 225, 236, 244,* ***258****,* ***279****,* ***280****, 282, 289, 291, 292, 298, 309, 312, 315,* ***328****, 329,* ***331****,* ***342****, 344, 357,* ***362****, 372,* ***377****, 392, 405,* ***408****, 410, 416, 418,* ***421****, 429, 448, 450, 453,* ***461****, 462, 470, 471, 479, 483* | ***44****, 48, 73, 108, 117, 172, 174, 189, 203, 205, 210, 213, 244, 282, 288, 291, 292, 309, 312, 318,* ***331****,* ***342****,* ***362****, 371, 405, 407, 447, 462, 467, 470, 471, 482* | ***44****, 47, 48, 53, 57, 63, 147,* ***175****, 210, 213, 225, 236, 244,* ***258****, 282, 291, 292, 309,* ***328****, 329,* ***331****, 344,* ***362****, 410, 450, 467* |
| Power analysis is reported | ***10****, 75,* ***82****, 85,* ***100****, 125,* ***157****, 174, 199, 221, 232, 248,* ***277****, 293, 309, 346,* ***358****, 375,* ***376****,* ***415****, 429,* ***446****,* ***456****,* ***485****,* ***495*** | ***10****, 75,* ***82****, 85,* ***100****, 125, 150,* ***157****, 174, 232, 276,* ***277****, 293, 309, 326, 346, 375,* ***415****,* ***417****,* ***446****, 468,* ***485*** | *85, 125, 130,* ***157****,* ***233****,* ***277****, 309, 346, 375,* ***415****,* ***446****, 468,* ***495*** |
| Replication study | *126, 139,* ***204****, 244, 273, 337, 339, 352,* ***413****, 457* | *113, 181,* ***204****, 244, 339, 457* | *126, 181,* ***204****, 244, 273, 337, 352, 457* |

Note: Numbers correspond to the COIS IDs in the COIS table. **Bolded** COIS ID numbers are experimental studies and un-bolded COIS ID numbers are observational studies.

Supplemental table 2: Unplanned exploratory analysis of citation counts and open science practices for observational studies only (*n* = 410)

|  | Citation Count | |  |  |  |
| --- | --- | --- | --- | --- | --- |
|  | Did not use practice  *M* (SD)  Median | Used  practice  *M* (SD)  Median | *t* | *p-*value | Cohen’s *d* |
| Any open science practice | 15.4 (17.2)  11.0 | 19.4 (25.4)  12.5 | -1.8717 | 0.0620 | — |
| Pre-registration | 17.5 (22.0)  11.0 | 13.5 (13.4)  13.5 | 0.4216 | 0.7445 | — |
| Open data | 17.1 (19.6)  11.0 | 32.3 (61.9)  15.5 | -0.8480 | 0.4144 | — |
| Open notebook | — | — | — | — | — |
| Open access | 16.0 (18.0)  11.0 | 20.4 (27.7)  13.0 | -1.7003 | 0.0906 | — |
| Open materials | 16.7 (18.5)  11.0 | 26.4 (45.3)  13.0 | -1.2130 | 0.2338 | — |
| Open code | 17.0 (19.4)  11.0 | 58.8 (94.1)  17.0 | -0.9926 | 0.3770 | — |
| Preprint | 16.3 (18.0)  11.0 | 24.6 (37.1)  15.0 | -1.6733 | 0.0992 | — |
| Power analysis is reported | 17.7 (22.3)  11.0 | 14.0 (12.6)  8.0 | 1.1551 | 0.2604 | — |
| Replication study | 17.5 (22.1)  11.0 | 16.9 (11.7)  13.0 | -0.1624 | 0.8740 | — |

Note: Table shows means, standard deviations (SD) and medians for any open science practice and each of the nine open science practices separately. All *t*-tests used Welch’s adjustment for unequal variances.

Supplemental table 3: Unplanned exploratory analysis of citation counts and open science practices for experimental studies only (*n* = 90)

|  | Citation Count | |  |  |  |
| --- | --- | --- | --- | --- | --- |
|  | Did not use practice  *M* (SD)  Median | Used  practice  *M* (SD)  Median | *t* | *p-*value | Cohen’s *d* |
| Any open science practice | 13.5 (11.0)  11.0 | 17.6 (16.5)  12.0 | -1.4387 | 0.1540 | — |
| Pre-registration | 15.9 (14.5)  12.0 | 21.0 (20.6)  13.0 | -0.6012 | 0.5722 | — |
| Open data | 16.1 (15.0)  12.0 | 18.0 (14.0)  14.0 | -0.2631 | 0.8080 | — |
| Open notebook | — | — | — | — | — |
| Open access | 14.2 (12.5)  11.5 | 19.5 (17.8)  15.0 | -1.5365 | 0.1304 | — |
| Open materials | 15.4 (13.9)  12.0 | 27.2 (24.2)  16.0 | -1.1735 | 0.2912 | — |
| Open code | 16.0 (14.9)  12.0 | 25.0 (18.4)  25.0 | -0.6872 | 0.6140 | — |
| Preprint | 16.6 (14.6)  12.5 | 14.3 (16.4)  7.5 | 0.5335 | 0.5995 | — |
| Power analysis is reported | 15.3 (13.9)  12.0 | 20.9 (19.4)  14.5 | -1.0322 | 0.3178 | — |
| Replication study | 24.5 (33.2)  24.5 | 16.0 (14.6)  12.0 | 0.3604 | 0.7794 | — |

Note: Table shows means, standard deviations (SD) and medians for any open science practice and each of the nine open science practices separately. All *t*-tests used Welch’s adjustment for unequal variances.
